# 4-Oxo-2-Nonenal- and Agitation-Induced Aggregates of α-Synuclein and Phosphorylated α-Synuclein with Distinct Biophysical Properties and Biomedical Applications

**DOI:** 10.3390/cells13090739

**Published:** 2024-04-24

**Authors:** Tie Wang, Weijin Liu, Qidi Zhang, Jie Jiao, Zihao Wang, Ge Gao, Hui Yang

**Affiliations:** 1 Department of Neurobiology, School of Basic Medical Sciences, Capital Medical University, Beijing 100069, China; wangtie198@163.com (T.W.); liuweijin@crrc.com.cn (W.L.); k729724031@icloud.com (Q.Z.); jiaojietongxue@ccmu.edu.cn (J.J.); 18301226@muc.edu.cn (Z.W.); 2 Center of Parkinson’s Disease, Beijing Key Laboratory of Neural Regeneration and Repair, Key Laboratory for Neurodegenerative Disease of the Ministry of Education, Beijing Institute of Brain Disorders, Beijing 100069, China

**Keywords:** α-synuclein, 4-oxo-2-nonenal (ONE), aggregates, preformed fibrils (PFF), phosphorylation, enzyme-linked immunosorbent assay

## Abstract

α-Synuclein (α-syn) can form oligomers, protofibrils, and fibrils, which are associated with the pathogenesis of Parkinson’s disease and other synucleinopathies. Both the lipid peroxidation product 4-oxo-2-nonenal (ONE) and agitation can induce aggregation of α-syn and phosphorylated α-syn. Thus, clarification of the characteristics of different α-syn species could help to select suitable aggregates for diagnosis and elucidate the pathogenesis of diseases. Here, we characterized ONE-induced wild-type (WT) α-syn aggregates (OW), ONE-induced phosphorylated α-syn (p-α-syn) aggregates (OP), agitation-induced α-syn preformed fibrils (PFF), and agitation-induced p-α-syn preformed fibrils (pPFF). Thioflavin T (ThT) dying demonstrated that OW and OP had fewer fibrils than the PFF and pPFF. Transmission electron microscopy revealed that the lengths of PFF and pPFF were similar, but the diameters differed. OW and OP had more compact structures than PFF and pPFF. Aggregation of p-α-syn was significantly faster than WT α-syn. Furthermore, OW and OP were more sodium dodecyl sulfate-stable and proteinase K-resistant, suggesting greater stability and compactness, while aggregates of PFF and pPFF were more sensitive to proteinase K treatment. Both ONE- and agitation-induced aggregates were cytotoxic when added exogenously to SH-SY5Y cells with increasing incubation times, but the agitation-induced aggregates caused cell toxicity in a shorter time and more p-α-syn inclusions. Similarly, p-proteins were more cytotoxic than non-p-proteins. Finally, all four aggregates were used as standard antigens to establish sandwich enzyme-linked immunosorbent assay (ELISA). The results showed that the recognition efficiency of OW and OP was more sensitive than that of PFF and pPFF. The OW- and OP-specific ELISA for detection of p-α-syn and α-syn in plasma samples of Thy1-α-syn transgenic mice showed that the content of aggregates could reflect the extent of disease. ONE and agitation induced the formation of α-syn aggregates with distinct biophysical properties and biomedical applications.

## 1. Introduction

Abnormal fibrillation and aggregation of α-synuclein (α-syn) are considered major pathological hallmarks of Parkinson’s disease (PD), dementia with Lewy bodies, and multiple system atrophy [1]. Lewy body inclusions in PD contain a significant fraction of aggregated α-syn, of which 90% is phosphorylated at serine-129 (Ser129) [2]. Many recent studies have attempted to unravel the mechanisms of α-syn aggregation and fibril formation in order to clarify the relationship between these processes and neuronal death [3]. Accumulating evidence suggests that Ser129 phosphorylation is a representative hallmark of the fibrillation process, which increases the levels of toxic α-syn aggregates [4,5,6]. Previous experiments have validated, coupled with human biofluids and tissue samples, the pathogenic significance of aggregative species in triggering and aggravating α-syn aggregation in PD [7,8]. As early biomarkers of PD, α-syn and p-α-syn aggregates are pathogenic [9]. Hence, detection of α-syn and p-α-syn aggregates are useful as early diagnostic and prognostic biomarkers.

A major obstacle to elucidating the role of α-syn pathology has been the process of isolating these aggregates in a stable form to study the forms of oligomers and potential impacts on neuronal function. Therefore, in addition to effects on neurodegeneration, clarification of the polymorphisms of α-syn aggregates, including the connection between heterogeneity and toxicity, is crucial for early diagnosis, prognosis, and drug development. Moreover, neurons contain high contents of polyunsaturated fatty acids that when peroxidated form highly reactive aldehydes [10], including 4-oxo-2-nonenal (ONE), 4-hydroxyl-2-nonenal (HNE), malondialdehyde, and acrolein [11,12], which all exhibit considerable cytotoxicity of oxidative stress and can modify proteins. A characteristic of PD is the oxidative stress that results in the formation of aldehydes by lipid peroxidation, which promote α-syn aggregation [13]. ONE is more efficient than HNE at inducing α-syn aggregates [14]. ONE-induced oligomers are rich in β-sheet structure and are cytotoxic in neuroblastoma (SH-SY5Y) cells [15]. ONE-α-syn aggregates have been characterized and shown to have similar size, morphology, and toxicity as unmodified α-syn aggregates; however, ONE is able to cross-link, making the ONE- aggregates much more stable [14]. In a model focusing on the misfolded, aggregated forms of α-syn as the PD model, recombinant α-syn monomeric proteins are agitated under defined conditions in vitro to generate aggregated, amyloid pre-formed fibrils (PFFs) that are similar in structure to the building blocks of Lewy bodies and Lewy neurites [16]. PFF, synthesized from recombinant α-syn by physical shaking, are widely used as an in vitro model for aggregation, propagation, and hyperphosphorylation of endogenous phosphorylated α-syn (p-α-syn) [17,18,19]. Although the PFF generated from recombinant α-syn induces a model using supra-physiological levels of α-syn, the levels of α-syn are much closer to that of the human condition as compared to viral vector-based and some transgenic models [16]. PFF could initiate the conversion of normal endogenous proteins to a pathogenic hyper-phosphorylated at Ser129 aggregated form of α-syn [17]. However, the effect of the phosphorylation of α-syn aggregates in the seeding and pathological accumulation of α-syn has not been examined.

However, thus far, no study has compared the properties of α-syn and p-α-syn aggregates induced by ONE and physical shaking. Therefore, we assessed their structural, biophysical, and functional properties by a combination of thioflavin-T (Th-T) fluorescence, electron microscopy, toxicity in cells, and their usability as calibrators in ELISA.

## 2. Materials and Methods

### 2.1. Cloning, Expression, and Purification of Recombinant Human α-Syn

The expression and purification of α-syn has been described elsewhere [20]. Briefly, competent BL21 (DE3) cells (CB105-01; Tiangen Biotech Co., Ltd., Beijing, China) were transformed with the pGEX-4T-1 expression vector coding for full-length human α-syn. Glutathione S-transferase-tagged α-syn was expressed after induction with 0.1 mM isopropyl-β-D-1-thiogalactopyranoside. The purified recombinant protein was extracted from the bacterial lysate by combining with glutathione SepharoseTM 4B resin (GE Healthcare Life Sciences, Chicago, IL, USA). α-Syn was collected after digestion with human thrombin for 6 h at room temperature. The elution buffer of α-syn was discarded by filtration (cut-off, 10 kD; EMD Millipore Corporation, Billerica, MA, USA) and replaced with a working buffer (20 μM HEPES, 10 μM MgCl_2_, 20 μM dithiothreitol, pH 7.4). The purity of the collected α-syn was assessed by western blot (WB) analysis. The protein concentration of α-syn was measured with the bicinchoninic acid protein assay (Thermo Fisher Scientific, Waltham, MA, USA). Aliquots of α-syn were stored at −80 °C.

### 2.2. Preparation of p-α-Syn

Polo-like-kinase 3 (PLK3, Sigma-Aldrich Corporation, St. Louis, MO, USA) is reported to phosphorylate α-syn at Ser129 in vitro [21]. Phosphorylation of α-syn at Ser129 was performed as previously described [20]. After incubation for 3 h in a reaction buffer containing 50 μL of α-syn (2 mg/mL), 1.5 μL of PLK3 (1.44 mg/mL) and 0.7 μL of adenosine triphosphate (Sigma-Aldrich Corporation, St. Louis, MO, USA) at 30 °C, the reaction was terminated by the addition of 25 mM ethylenediaminetetraacetic acid disodium salt. The p-α-syn protein was verified by WB analysis with an antibody (Ab) against p-Ser129 of α-syn (pSyn#64; Wako Pure Chemical Industries, Ltd., Osaka, Japan). The phosphorylation state of α-syn was further confirmed by mass spectrometry (supported by the Proteomics platform, Institute of Genetics and Developmental Biology, Chinese Academy of Sciences, Beijing, China). The p-α-syn monomer was stored at −80 °C.

### 2.3. Preparation of ONE-Induced Wild-Type (WT) α-Syn Aggregates (OW), ONE-Induced p-α-Syn Aggregates (OP), Agitation-Induced α-Syn PFF, and p-α-Syn Preformed Fibrils (pPFF)

**Scheme 1:**
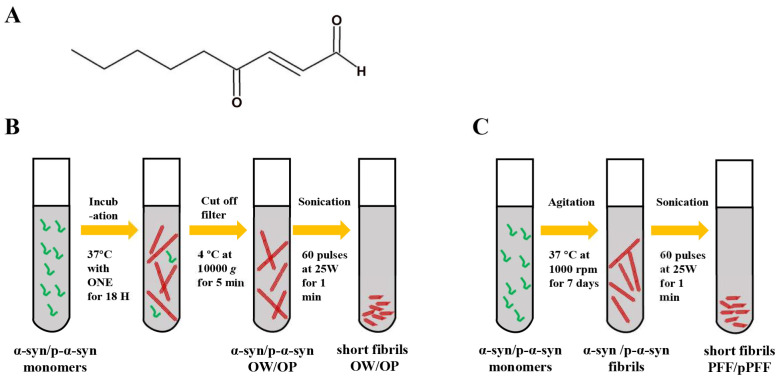
Preparation of α-syn and p-α-syn aggregates by ONE and agitation. (**A**) Structural formula of ONE. (**B**) Incubation of α-syn and p-α-syn monomers with ONE for 18 h formed OW and OP. (**C**) Agitation of α-syn and p-α-syn monomers at 37 °C and 1000 rpm for 7 days induced α-syn PFF and p-α-syn pPFF. The aggregates were sonicated at 25 W for 1 min in 60 pulses before treating cells.

As depicted in Scheme 1A, 1.5 µL of ONE (10 mg/mL; Cayman Chemicals, Ann Arbor, MI, USA) were added to 50 µL of monomeric α-syn/p-α-syn (2 mg/mL) and incubated for 18 h at 37 °C in a horizontal shaker to prepare the OW and OP. The non-reacted ONE was removed by filtration (cut-off, 10 kD; EMD Millipore Corporation) and centrifugation at 4 °C and 10,000× *g* for 5 min (Scheme 1B). A microcentrifuge tube containing 100 μL of α-syn/p-α-syn (4 mg/mL) was sealed and incubated at 37 °C under constant agitation at 1000 rpm on an Eppendorf ThermoMixer^®^ (Eppendorf AG, Hamburg, Germany) for 7 days to prepare the PFF and pPFF (Scheme 1C). The aggregates were aliquoted and stored at −80 °C. The aggregates were sonicated at 25 W for 1 min in 60 pulses to form short aggregates for further use.

### 2.4. Thioflavin T (ThT) Staining

Monomeric α-syn, p-α-syn, and ONE- and agitation-induced aggregates (OW, OP, PFF, and pPFF, 8 μM) were incubated in staining solution (20 mM Tris, pH 7.4, 0.15 M NaCl, 19 μM ThT [Sigma-Aldrich Corporation, St. Louis, MO, USA]) at 37 °C in the wells of a nonbinding polystyrene 96-well plate (PerkinElmer, Waltham, MA, USA). Fluorescence was measured every 60 min at excitation and emission wavelengths of 440 and 485 nm, respectively. All samples were tested in triplicate. Background fluorescence was measured and deducted.

### 2.5. Transmission Electron Microscopy (TEM)

Morphological differences of α-syn, p-α-syn, OW, OP, PFF, and pPFF were observed by TEM. The samples were diluted to 20 μg/mL with 0.01 M phosphate-buffered saline (PBS) and applied onto 200-mesh copper grids (Agar Scientific, Stansted, Essex, UK), which were precoated with Formvar. After 6 min, the grids were blotted with filter paper, negatively stained with 5% uranyl acetate for 3 min, blotted, and dried under infrared radiation for 24 h at 37 °C. The protein structures were observed with an electron microscope (JEM-2100; JEOL, Ltd., Tokyo, Japan).

### 2.6. Digestion with Proteinase K (PK)

Samples with 4 μg of OW, OP, PFF, and pPFF were incubated at 37 °C in PK solution (1 μg/mL of PK [Sigma-Aldrich Corporation, St. Louis, MO, USA] in 20 mM Tris, pH 7.4, 0.15 NaCl) for 0, 5, 10, 20, and 40 min. The enzymatic reaction was terminated by the addition of sample buffer containing sodium dodecyl sulfate (SDS) and dithiothreitol and then boiled at 95 °C for 5 min. Afterward, the proteins were separated by a SDS-Tris 12% gel and transferred to a polyvinylidenedi fluoride (PVDF) membrane (Millipore). Finally, α-syn and p-α-syn was visualized in an Odyssey imaging system (Li-Cor, Lincoln, NE, USA) using an α-syn (ab138501; Abcam, Cambridge, MA, USA) and p-α-syn (pSyn#64; Wako Pure Chemical Industries, Ltd.) monoclonal antibody followed by a secondary antibody (926-68070, 926-68071; dilution, 1:10,000; LI-COR Biosciences, Lincoln, NE, USA). Finally, the membranes were analyzed using an Odyssey imaging system (Li-Cor).

### 2.7. WB Analysis

WB analysis was performed as previously described [20]. Briefly, samples of recombinant proteins (α-syn, p-α-syn, OW, OP, PFF, pPFF, 200 ng/lane) were mixed with loading buffer (250 mM Tris-HCl, pH 6.8, 30% glycerol, and 0.02% bromophenol blue), boiled at 95 °C for 5 min, separated by electrophoresis with 12% polyacrylamide gels, and then electroblotted at 2 mA/cm^2^ for 90 min to polyvinylidene difluoride membranes (EMD Millipore Corporation), which were incubated with 0.4% paraformaldehyde for 30 min, washed three times with tris-buffered saline/Tween 20 (TBST), blocked with 5% skim milk for 1 h at room temperature, and then probed overnight at 4 °C with Abs against α-syn (ab138501; Abcam, Cambridge, MA, USA) and p-α-syn (pSyn#64; Wako Pure Chemical Industries, Ltd.). It was then washed three times with TBST, incubated for 1 h with fluorophore-conjugated goat anti-mouse or anti-rabbit secondary Abs immunoglobulin G (IgG) (926-68070, 926-68071; dilution, 1:10,000; LI-COR Biosciences, Lincoln, NE, USA), and washed three times with TBST. The protein bands were visualized with an Odyssey imaging system (LI-COR Biosciences).

### 2.8. Cell Culture, Immunofluorescence, and Confocal Microscopy

Neuroblastoma (SH-SY5Y) cells were cultured in Dulbecco’s modified Eagle’s medium (Sigma-Aldrich Corporation) supplemented with 10% fetal bovine serum (Invitrogen Corporation, Carlsbad, CA, USA) and 1% antibiotic (streptomycin and penicillin) at 37 °C under an atmosphere of 5% CO_2_/95% air. For immunohistochemical analyses, the cells were fixed with 4% paraformaldehyde for 30 min, washed three times with PBS, permeabilized with 0.3% TritonX-100 in 0.01 M PBS for 10 min at room temperature, incubated for 60 min in 10% goat serum (5424; Cell Signaling Technology, Inc., Danvers, MA, USA), followed by incubation overnight at 4 °C with an Ab against p-α-syn (ab51253; Abcam, Cambridge, MA, USA), washed three times with PBST, incubated for 1 h at room temperature with a secondary Alexa Fluor Plus 594-conjugated polyclonal goat anti-rabbit Ab (A11037; Invitrogen Corporation), counterstained with 4′,6-diamidino-2-phenylindole for 5 min, and imaged with a confocal microscope (TCS 473 SP8; Leica Microsystems GmbH, Wetzlar, Germany). The experiment was repeated three times, with four fields of view randomly selected on each occasion. We quantified the extent of inclusion in each field.

### 2.9. Cell Viability Assay

Cell viability was determined with the thiazolyl blue tetrazolium bromide (MTT) assay as previously described [22]. Briefly, 1 × 10^4^ cells were seeded in the wells of a 96-well microplate and cultured with α-syn, p-α-syn, sonicated OW, OP, PFF, and pPFF for 1, 3, and 6 h. Afterward, the medium was replaced with MTT (M2128; Sigma-Aldrich Corporation) at a final concentration of 0.5 mg/mL and incubation was continued for 4 h. Then, the medium was discarded and replaced with 100 μL of dimethyl sulfoxide to dissolve the formazan crystals. Absorbance was measured at 490 nm with a microplate reader (PerkinElmer, Inc., Waltham, MA, USA). Three independent experiments were performed.

### 2.10. Mice

Transgenic (Tg) mice overexpressing human α-syn (017682; Jackson Laboratory, Bar Harbor, ME, USA) were housed in the Laboratory Animal Center of Capital Medical University (Beijing, China) at room temperature under a 12:12-h light: dark cycle. The protocol of the animal study was approved by the Institutional Animal Care and Use Committee of Capital Medical University (approval no. AEEI-2020-017) and conducted in accordance with the Guide for the Care and Use of Laboratory Animals (https://www.ncbi.nlm.nih.gov/books/NBK54050/ (accessed on 3 March 2024)).

### 2.11. Open Field Test

The open field test was used to analyze motor functioning in mice as previously described [23]. Briefly, the test uses a camera to measure movement of the test animal in the 50 × 50 × 30 cm black box, and peripheral zones of four small squares in the middle. When the timer is started, the mouse explores the test area for 5 min. The movement distance and speed were recorded in 5 min.

### 2.12. Rotarod Test

The rotarod test was used to analyze the motor function of 12-month-old mice as previously described [24]. Briefly, mice were trained once per day for 2 days with increasing rotation speeds of 4–40 rpm for 5 min. The time that the mice remained on the rotarod over the next 3 days was recorded and averaged.

### 2.13. Aggregates as Calibrators in Enzyme-Linked Immunosorbent Assay (ELISA)

As described previously [25], a sandwich ELISA was used to prepare standard curves to quality plasma proteins with OW, OP, PFF, and pPFF as standard antigens. Briefly, a 96-well microplate (Nunc Max iSorb, NUNC) was coated (50 μL/well) overnight at 4 °C with 0.2 g/mL polyclonal Ab against the N-terminus of α-syn (SN16; produced in our lab) in 200 mM of sodium bicarbonate buffer with pH 9.6. The plate was then washed with PBST (PBS containing 0.05% Tween-20), blocked with a blocking buffer (5% BSA in PBST) at 100 μL/well for 2 h at 37 °C, and then washed with PBST. The aggregates were prepared as standard by diluting them in PBS and then added to the plate (50 μL/well) and incubated for 2 h at 37 °C with shaking (40 rpm). After washing the plate with PBST, Abs against the C-terminus of human α-syn (ab138501; Abcam) and p-α-syn (C140S; produced in our lab) [20] were added to the plate (50 μL/well) and incubated for 2 h at 37 °C. After washing, alkaline phosphatase (AP)-conjugated goat anti-rabbit secondary Abs against IgG (SA00002-2; dilution, 1:1000; Proteintech, Wuhan, China) was added to each well and the plate was incubated at 37 °C for 1 h. After washing three times, 100 µL of enzyme-substrate p-nitrophenyl phosphate (N1891; Sigma-Aldrich Corporation) was added to each well to react with AP-IgG. The reaction was allowed to proceed for 30 min at 37 °C in the dark. Absorbance at 405 nm was measured with a microplate reader (Multiskan MK3; Thermo Fisher Scientific). Peripheral plasma samples of 60 12-month-old WT mice and Thy-α-syn Tg mice were diluted with PBST to 1:4000. Plasma levels of α-syn and p-α-syn aggregates were measured by the OW- and OP-specific standard curves.

### 2.14. Statistical Analysis

All statistical analyses were performed using Prism 8.0.1 software (GraphPad Software, Inc., San Diego, CA, USA). The results are presented as the mean ± standard deviation (SD) of at least three independent experiments. The significance of differences between two groups was determined with the unpaired two-tailed Student’s t-test and among three or more groups with one- or two-way analysis of variance (ANOVA) with Tukey’s or Sidak’s multiple comparison test. ImageJ software java 1.8.0 (https://imagej.net/ij/ (accessed on 3 March 2024)) was used to quantify pixel intensities. A probability (*p*) value < 0.05 was considered statistically significant.

## 3. Results

### 3.1. ONE- and Agitation-Induced Formation of α-Syn and p-α-Syn Aggregates with Distinct Morphologies and Structures

**Figure 1:**
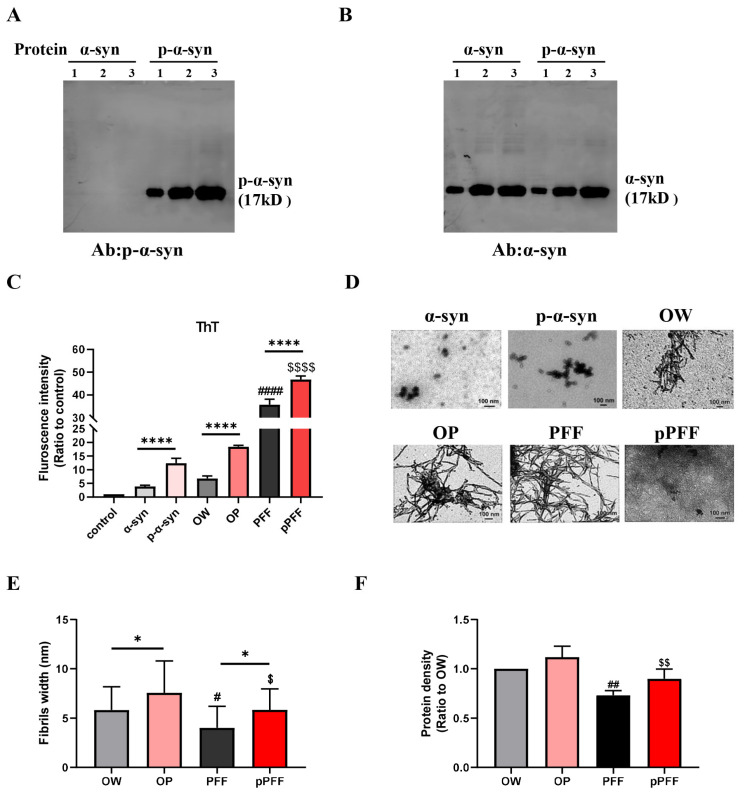
OW, OP, PFF, and pPFF had distinct morphological and structural properties. (**A**,**B**) WB analysis of the purified recombinant α-syn and p-α-syn with protein-specific Abs. Three gradient elution with low, moderate, and high concentrations (lanes 1, 2, and 3, respectively). (**C**) PFF and pPFF exhibited higher ThT signals than OW and OP (^####^ *p* < 0.0001 vs. OW, ^$$$$^ *p* < 0.0001 vs. OP). Furthermore, ThT signals of p-α-syn, OP, and pPFF were each much higher than α-syn, OW, and PFF (**** *p* < 0.0001). (**D**) Representative TEM images of α-syn, p-α-syn, OW, OP, PFF, and pPFF. (**E**) OW, OP, PFF, and pPFF had different fibril widths (* *p* < 0.05 vs. OW or OP, ^#^ *p* < 0.05 vs. OW, ^$^ *p* < 0.05 vs. OP). (**F**) TEM gray results showing that OW and OP exhibited more compact structures than PFF (^##^ *p* < 0.01 vs. OW) and pPFF (^$$^ *p* < 0.01 vs. OP). The results are expressed as the mean ± SD (one-way ANOVA, Tukey’s multiple comparisons test, n = 4). Scale bar = 100 nm.

The WB results showed that the p-α-syn and α-syn recombinant proteins (17 kD) at different concentrations could be specifically recognized by Abs against p-α-syn and α-syn (Figure 1A,B). After obtaining the monomeric α-syn and p-α-syn proteins, four different aggregate proteins were prepared in vitro with the use of ONE (OW and OP) and agitation (PFF and pPFF), respectively. The fluorescence intensity of the dye ThT increases upon binding to β-sheet structures. Although both aggregate forms showed increased ThT signals, compared to the native monomeric protein, PFF and pPFF exhibited higher ThT signals than OW (*p* < 0.0001) and OP (*p* < 0.0001). Furthermore, the ThT signals of p-α-syn, OP, and pPFF were much higher compared to α-syn, OW, and PFF (*p* < 0.0001 vs. non-p-α-syn) (Figure 1C). These findings confirmed that OW, OP, PFF, and pPFF had polymerized into amyloid-like fibrils.

TEM analysis was conducted for morphological analysis of the α-syn aggregates. OW and OP appeared as relatively large amorphous round species, while PFF and pPFF had more protofibril-like structures. Monomeric α-syn and p-α-syn had more homogeneous appearances as small round structures (Figure 1D). ImageJ analysis showed that the fibril widths of OW and OP were greater than that of PFF (*p* < 0.05) and pPFF (*p* < 0.05), while OP and pPFF had larger widths than OW and PFF (*p* < 0.05) (Figure 1E). The intensity of OW and OP was higher than that of PFF (*p* < 0.01) and pPFF (*p* < 0.01) (Figure 1F).

### 3.2. ONE- and Agitation-Induced Formation of α-Syn and p-α-Syn Aggregates at Distinct Speeds

**Figure 2:**
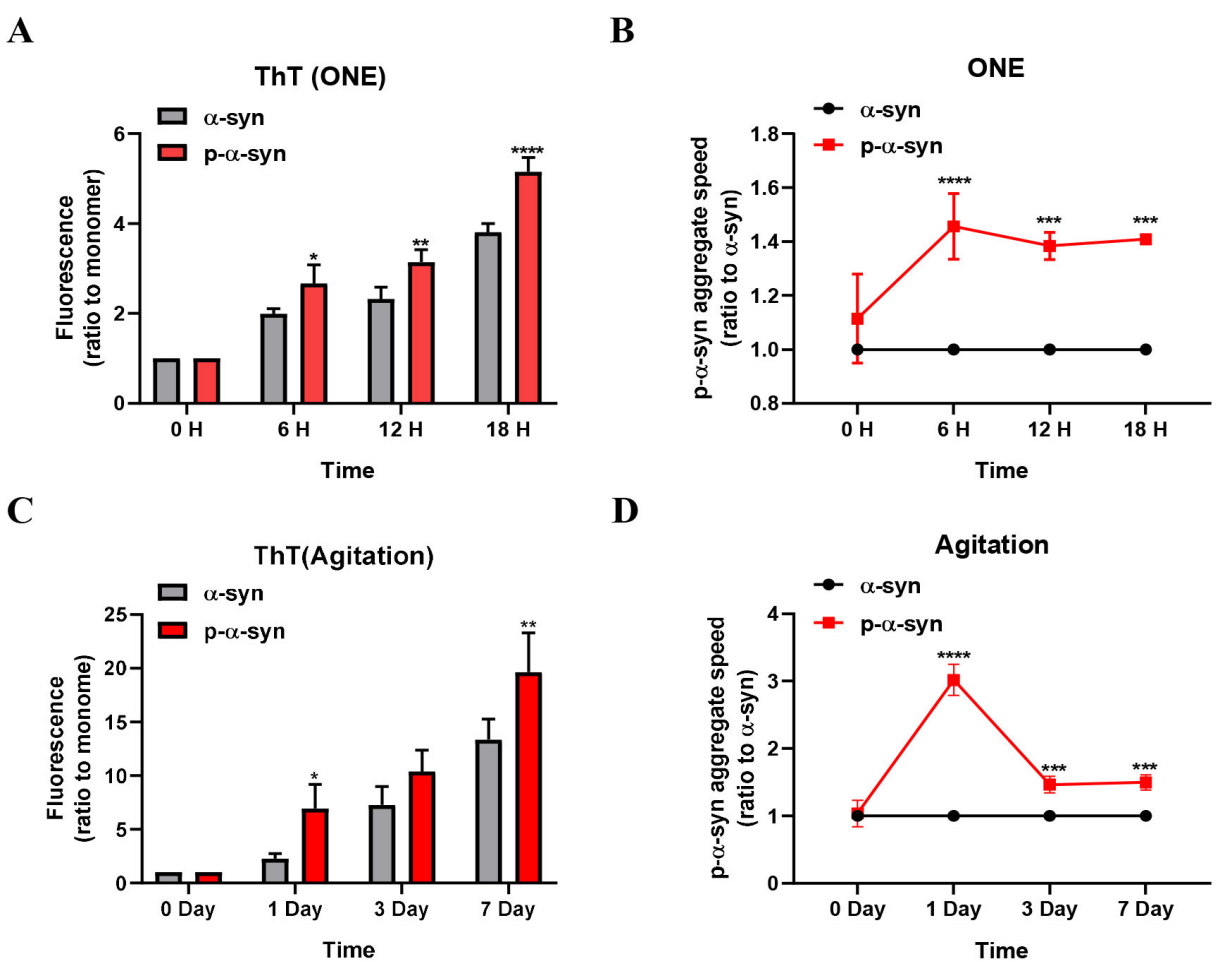
Aggregation rate of p-α-syn was faster than that of α-syn in ONE- and agitation-induced aggregates by ThT analysis. (**A**) The fluorescence intensity of p-α-syn aggregates induced by ONE was higher than that of α-syn aggregates at 6, 12, and 18 h. (**B**) The aggregation speed of ONE-induced p-α-syn was faster than that of α-syn. (**C**) ThT analysis detected fibrils formed gradually with shaking on days 1, 3, and 7. (**D**) Aggregation speed of agitationi-induced p-α-syn was faster than that of α-syn. The results are expressed as the mean ± SD (n = 4). * *p* < 0.05, ** *p* < 0.01, *** *p* < 0.001, **** *p* < 0.0001 vs. α-syn.

To verify the effect of phosphorylation on the aggregation rate induced by compound induction and physical shaking, the fluorescence intensity of ThT was measured at different incubation times. Although the ThT signals of OW and PFF increased with the incubation time, those of OP (6, 12, and 18 h) and pPFF (1, 3, and 7 days) were significantly greater. The ThT fluorescence intensity of both ONE- and agitation-induced p-α-syn aggregates were greater than that of α-syn aggregates (*p* < 0.05, 0.01 or 0.0001 vs. α-syn) (Figure 2A,C). OP and pPFF formed aggregates more rapidly than OW and pPFF at different incubation times (*p* < 0.001 or 0.0001 vs. α-syn) (Figure 2B,D).

### 3.3. Stability and Compactness of ONE- and Agitation-Induced α-Syn and p-α-Syn Aggregates

**Figure 3:**
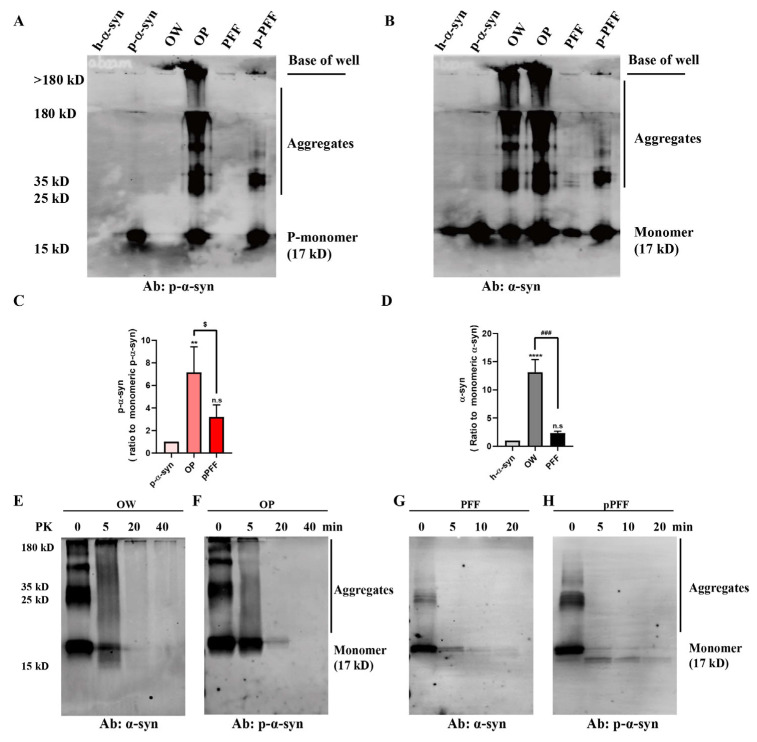
OW and OP exhibited different SDS stability and PK resistance with PFF and pPFF. (**A**,**B**) A band exceeding 180 kD that had not entered the stacking gel of the SDS-treated OP and OW. In addition, a strongly labeled high-molecular-weight smear (17–180 kD) was detected (**A**) OP; (**B**) OW). In contrast, SDS-treated pPFF and PFF dissociated into a narrow range of low-molecular-weight species. Moreover, a faintly high-molecular-weight smear (>180 kD) was also observed (**A**) pPFF; (**B**) PFF. (**C**) Quantification of the p-α-syn in OP and pPFF. (**D**) Quantification of α-syn in OW and PFF. (**E**) A band exceeding 180 kD that had not entered the stacking gel could be observed in OW and OP. A band exceeding 180 kD and a faint high-molecular-weight smear (25–180 kDa) were observed at 5 min PK treatment and no bands at 20 min in the OW group. (**F**) The phosphorylated protein bands disappeared after PK treatment for 40 min in OP. (**G**) No bands were detected after 10 min PK treatment in OW. (**H**) No bands were detected after 20 min PK treatment in OP. The results are expressed as mean ± SD (n = 3). ^$^ *p* < 0.05 vs. p-α-syn, ^###^ *p* < 0.001 vs. α-syn, ** *p* < 0.01 vs. p-α-syn, **** *p* < 0.0001 vs. α-syn, n.s., not significant.

The stability of ONE- and agitation-induced α-syn aggregations was assessed by Western Blot analysis. A strongly labeled band of OP exceeding 180 kD that did not enter the stacking gel was observed (above the base of well). Of the OP samples, aggregate bands of α-syn ranging in size between 25 and 180 kD were smeared. The bands of pPFF were faint, indicating low-molecular weight ranging in size between 25 and 50 kD (Figure 3A). Quantification of the WB results showed that the p-α-syn aggregates (>17 kD) of the OP samples was significantly greater than in the pPFF groups (*p* < 0.01) (Figure 3C). The membranes were also probed with an Ab against α-syn. The OW samples showed strong aggregation compared to the pPFF samples, ranging in size from 25 to 180 kD, with one band >180 kD. The pPFF samples only showed slight aggregation at 25–35 kD (Figure 3B). The α-syn aggregates (>17 kD) was significantly higher in the OW samples than the PFF samples (*p* < 0.0001) (Figure 3D). Strongly labeled monomeric bands were observed in all groups. These results suggest that the OW and OP were more stable than the PFF and pPFF under the same SDS conditions.

The compactness of the ONE- and agitation-induced aggregations treated with PK was assessed by Western Blot analysis. A band exceeding 180kD that had not entered the stacking gel could be observed in OW and OP (Figure 3E, Line 0 and Figure 3F, Line 0). A band of monomers (17 kD) and a faint high-molecular-weight smeared bands of aggregates (100–180 kD) were observed after 5 min of PK treatment (Figure 3E Line 5 and Figure 3F Line 5). A faint band was detected at 20 min in OP (Figure 3F, Line 20), whereas almost no protein bands were detected in OW (Figure 3E, Line 20). In contrast, only a faint monomer band of the PFF and pPFF was observed after PK treatment for 5 min, while there were no high-molecular-weight smeared bands (Figure 3G, Line 5 and Figure 3H, Line 5). Differently, a faint truncated band of pPFF was observed at 10 and 20 min (Figure 3H, Line 10 and 20). These results indicate that OW and OP were more resistant to PK than PFF and pPFF, and the p-α-syn aggregates were more resistant than the non-p-α-syn aggregates.

### 3.4. ONE- and Agitation-Induced Aggregates Reduced Cell Viability and Formed p-α-Syn Inclusions to Different Degrees

**Figure 4:**
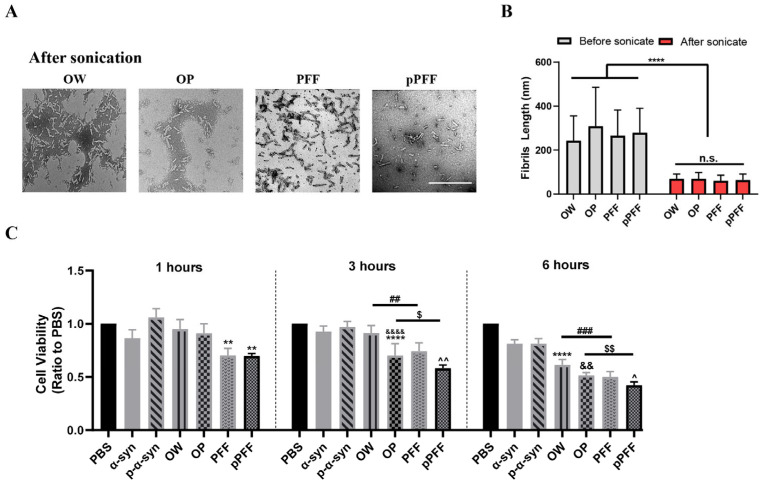
MTT results demonstrated that both PFF and pPFF exhibited a greater propensity to induce a reduction in cell viability compared to OW and OP. (**A**) Representative TEM image showing the morphologies of sonicated OW, OP, PFF, and pPFF. (**B**) Quantification of aggregate lengths before and after sonication. OW, OP, PFF, and pPFF had similar fibrils length after sonication. (**C**) The PFF and pPFF significantly reduced cell viability at 1 h compared to PBS and monomeric α-syn and monomeric p-α-syn. OP decreased cell viability at 3 h and OW decreased cell viability at 6 h. At 3 and 6 h, ONE- and agitation-induced p-aggregates and non-p-aggregates decreased cell viability with the incubation time. No significant difference in viability was observed in cells treated with monomeric α-syn and p-α-syn. The data are expressed as mean ± SD (one-way ANOVA, Tukey’s multiple comparisons test, n = 3). ** *p* < 0.01, **** *p* < 0.0001 vs. PBS. ^##^ *p* < 0.01, ^###^ *p* < 0.001 vs. OW. ^$^ *p* < 0.05, ^$$^ *p* < 0.01 vs. OP. ^&&^ *p* < 0.01, ^&&&&^ *p* < 0.0001 vs. OW. ^ *p* < 0.05, ^^ *p* < 0.01 vs. PFF. Scale bar = 200 nm.

Previous studies have suggested that ONE modification and agitation-induced fibrils can be taken up by cells [3,26]. To dynamically investigate the toxic properties of ONE- or agitation-induced aggregates, the MTT assay was used to evaluate the viability of SH-SY5Y neuroblastoma cells exposed to aggregates of sonicated OW, OP, PFF, and pPFF. TEM analysis showed that after sonication, the lengths of OW, OP, PFF, and pPFF had significantly decreased (*p* < 0.0001). The lengths of the fibrils ranged from 60.25 ± 25.42 to 69.75 ± 21.23 nm (Figure 4A,B). After incubation for 1 h, PFF and pPFF decreased cell viability compared to the control, while the amount of OP had significantly decreased at 3 h. After incubation for 6 h, the viability of the OW group had significantly decreased. Both at 3 h and 6 h, cell viability was lower in the OP and pPFF groups than in the OW and PFF groups, respectively. (Figure 4C).

**Figure 5:**
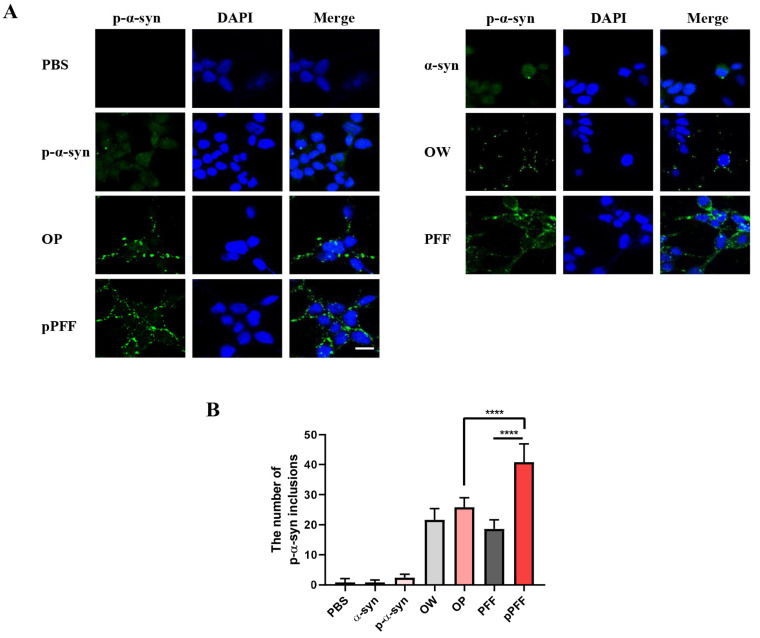
OW, OP, PFF, and pPFF facilitated the formation of p-α-syn inclusions to varying degrees in the SH-SY5Y neuroblastoma cell line. (**A**) Immunofluorescence staining of p-α-syn (green) in SH-SY5Y cells showed that monomeric α-syn, p-α-syn, and sonication of OW, OP, PFF, and pPFF for 12 h had different effects on p-α-syn granular aggregates or inclusions. (**B**) Quantitative analysis showed pPFF had induced more p-α-syn inclusions than PFF, OP, and OW. The data are expressed as the mean ± SD of three independent experiments. **** *p* < 0.0001. Green, p-α-syn; blue, nuclei. Scale bars = 20 um.

Consistent with results of the MTT assay, the immunofluorescence results showed widely distributed cytosolic p-α-syn granular aggregates or inclusions in the OW, OP, PFF, and pPFF groups. Monomeric α-syn, p-α-syn, had induced few p-α-syn inclusions (Figure 5A). The pPFF group had more p-α-syn inclusions than the PFF, OP, and OW groups (*p* < 0.0001). (Figure 5B).

### 3.5. ONE- and Agitation-Induced Aggregates Could Be Used as Standard Antigens for the Sandwich ELISA in Different OD Values

**Figure 6:**
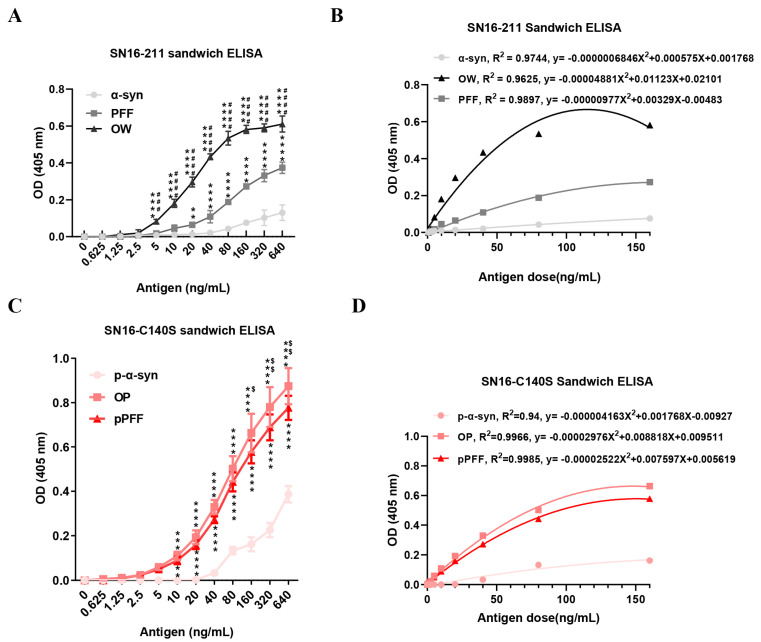
OW and OP had higher detection sensitivities compared to PFF and pPFF when utilized in sandwich ELISA. (**A**) Detection of α-syn, OW, and PFF using SN16-211 double-antibody sandwich ELISA with different antigen concentration. (**B**) Standard curve for detection of α-syn aggregates established from (**A**). (**C**) Detection of p-α-syn, OP, and pPFF using SN16-C140S double-antibody sandwich ELISA with different antigen concentrations. (**D**) Standard curve for detection of α-syn aggregates established from (**C**). Absorbance was measured at 405 nm. SN16, anti-α-syn N-terminal polyclonal Ab; 211, anti-α-syn C-terminal monoclonal Ab; C140S, anti-p(Ser129)-α-syn Ab. The results are expressed as the mean ± SD. ** *p* < 0.01, *** *p* < 0.001, **** *p* < 0.0001 vs. α-syn or p-α-syn. ^###^ *p* < 0.001, ^####^ *p* < 0.0001 vs. PFF. ^$^ *p* < 0.05, ^$$^ *p* < 0.01 vs. pPFF.

Sandwich ELISAs were employed to assess the stabilities of OW, OP, PFF, and pPFF using different concentrations of aggregates as standard antigens. The optical density (OD) values of OW were significantly higher than that of the α-syn group and PFF with a standard antigen concentration from 5 ng/mL (*p* < 0.0001 vs. α-syn, *p* < 0.001 vs. PFF), while the OD value of PFF had increased from 20 ng/mL (*p* < 0.01 vs. α-syn) (Figure 6A). The standard curves of OW and PFF had similar R2 values (Figure 6B). Similarly, the OD values of OP and pPFF had increased compared to the OD value of p-α-syn from 10 ng/mL (*p* < 0.001, *p* < 0.01). Meanwhile, the OD value of OP had increased from 160 ng/mL (*p* < 0.05 vs. pPFF) (Figure 6C). The standard curves of OW and OP had similar R2 values (Figure 6D).

### 3.6. The Detection of Aggregates α-Syn and p-α-Syn in the Plasma of Transgenic Mice Using OW- and OP -Special Sandwich Elisa Assay Demonstrated Concordance with the Observed Motor Dysfunction in These Mice

**Figure 7:**
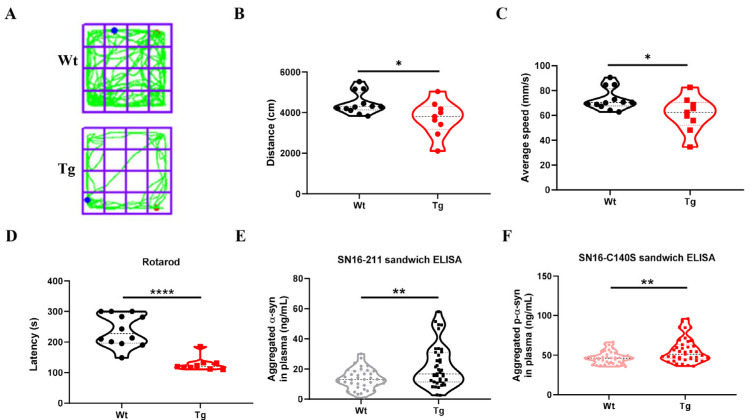
The levels of α-syn and p-α-syn aggregates in mice plasma by OW- and OP-specific ELISA were consistent with the motor dysfunction. (**A**) The motor track, (**B**) motor distance and (**C**) average speed showed the motor function of 12-month-old Wt and Tg mice using an open field test (* *p* < 0.05). (**D**) The rotarod test was used to assess motor function (**** *p* < 0.0001). (**E**) Concentrations of α-syn aggregates in the plasma of Tg mice plasma were significantly higher in Wt mice using the OW-specific ELISA. (**F**) Concentrations of p-α-syn aggregates in the plasma of Tg mice plasma were significantly higher than in Wt mice using the OP-specific ELISA (n = 30). Absorbance was measured at 405 nm. The results are expressed as the mean ± SD (unpaired *t*-test). ** *p* < 0.01 vs. WT.

Therefore, the OW- and OP-specific ELISAs were used to measure the plasma Thy1-α-syn levels of Tg mice. Motor behavior was evaluated with the open field and rotarod tests. The results of the open field test revealed that the Tg mice had moved shorter distances (Figure 7A,B) and at slower speeds (Figure 7C) than the WT mice (*p* < 0.05). Similarly, the Tg mice spent less time on the rotarod than the WT mice (*p* < 0.0001) (Figure 7D). These results indicate that Tg mice exhibited motor deficits. The results of the OW-specific ELISA showed that the plasma levels of α-syn aggregates were higher in the Tg mice than the WT mice (21.81 ± 2.252 vs. 13.37 ± 1.075 ng/mL, respectively, *p* < 0.01) (Figure 7E). Similarly, the results of the OP-specific ELISA showed that the plasma levels of p-a-syn aggregates were higher in the Tg mice than the WT mice (56.04 ± 2.506 vs. 46.85 ± 1.253 ng/mL, respectively, *p* < 0.01) (Figure 7F).

## 4. Discussion

Prefibrillar aggregates, such as oligomers and protofibrils, play central roles in various neurodegenerative diseases. The amphipathic N-terminus (1–60) possesses the α-helical propensity involved in membrane binding, and the hydrophobic central region (61–95) known as the non-amyloid-beta component (NAC) is rich in β-sheet structure responsible for aggregation and the acidic flexible C-terminus (96–140) [27]. α-Syn is a major component of cytoplasmic inclusions, such as Lewy bodies in PD and dementia with Lewy bodies, as well as glial cytoplasmic inclusions in multiple system atrophy [28,29]. Growing evidence suggests that the toxicity of α-syn would be limited to its aggregated form. Additionally, oligomeric forms of α-syn were identified and quantified in biological fluids, including serum and cerebrospinal fluid, as well as post-mortem brain tissues from patients with synucleinopathies [30,31,32,33,34]. Although these findings provide support for the involvement of α-syn aggregative species in neurodegeneration, the mechanisms underlying their toxicity remain unknown. Many approaches have been established to investigate the oligomeric state of α-syn in vitro. These α-syn aggregates were found to be mostly heterogeneous in nature with varying shapes and sizes that differed greatly based on the method of preparation [35]. Numerous preparations of α-syn aggregates have been documented to exhibit either toxic or non-toxic properties. However, the limited knowledge and understanding of the in vivo aggregates still pose a challenge in identifying which preparation truly resembles the in vivo-formed aggregative species. Here, we utilized lipid peroxidation metabolite ONE and agitation to generate α-syn aggregates, compared their properties using biochemical and biophysical methods, and verified their use as standard sample in an immunoassay. ONE, the major lipid peroxidation byproduct of poly-unsaturated fatty acids, was found to covalently modify α-syn and produce oligomers in vitro [36], has been suggested to reach the millimolar range in vivo [11,37], and reacts rapidly with the side chains of Arg, Cys, His, and Lys residues in proteins via Michael addition (Cys, His, or Lys residues) or through Schiff base formation (Lys residues) [38]. α-Syn contains one histidine residue, 15 lysine residues between amino acids 6 and 103, and no cysteine residue [39].

The use of PFF to induce endogenous α-syn to form pathological phosphorylation and trigger neurodegeneration is a popular model for studying PD and exploring therapeutic strategies. The advantages of this model are its ability to recapitulate the phosphorylation/aggregation of α-syn and nigrostriatal degeneration, as well as its suitability for studying the progressive nature of PD and the spread of α-syn pathology [35]. The addition of PFF synthesized from recombinant α-syn to neurons seeds recruitment of endogenous α-syn into aggregates is characterized by detergent insolubility and hyperphosphorylation. Hydrogen bonding and electrostatic, and hydrophobic interactions stabilize the β-structure of fibrillar amyloid aggregates [40,41]. α-Syn aggregation can occur in cell lines and many kinds of primary neurons in a PFF model [42,43,44], which could be used to elucidate how α-syn aggregates may spread among neurons [45,46].

OW and OP produced fibrils and middle ThT signals compared to PFF and pPFF (Figure 1). Partially consistent, a prior study showed that ONE-modified α-syn displayed low ThT signals and failed to fibrillate despite prolonged incubation times [10]. This difference could possibly be explained by the slightly higher molar ratio of ONE/α-syn used in the present study. Aggregation of α-syn into amyloid fibrils is a multistep process where monomeric protein gradually becomes converted into various oligomeric forms before forming matured fibrils [47]. The ThT and TEM results showed the high content aggregates in OW, OP, PFF, and pPFF. Therefore, we only detected fibrils, not oligomers, in the following tests.

Interestingly, malondialdehyde-Lys modifications of α-syn have been observed in the substantia nigra of PD patients, indicating that Lys residues are accessible for lipid peroxidation [48]. As we know, ThT is an optically-inactive fluorophore possessing high affinity toward amyloid fibrils [49]. Interesting, the ThT for OW and OP are not different from α-syn and p-α-syn, yet form fibrils (Figure 1D). Investigation of the amyloid fibril formation process requires not only the ability to distinguish the characteristic amyloid β-sheet structure from amorphous aggregates of the monomer or nonamyloid fibril forms of the precursor protein, but quantitation of the amyloid form as well [50]. PFF and ONE-induced aggregates have a different principle. ThT fluorescence intensity is not an absolute measure of the amount of formed amyloid (and indeed is not completely specific for amyloid folds). So, ThT could verify the fiber aggregation of PFF, but it could only partially reflect the fiber aggregation caused by ONE.

A detailed comparison of the speed of α-syn aggregation could help to select appropriate aggregate species for specific applications and research. ONE- and agitation-induced α-syn and p-α-syn aggregates formed amyloid-like fibrils with prolonged incubation (Figure 2A,C), implying that the four aggregates are thermodynamically stable. Some studies have shown that phosphorylation at Ser129 could affect the structure and aggregation of α-syn [51,52]. In this study, p-α-syn formed aggregates more rapidly than α-syn under the same conditions, which was consistent with the TEM results (Figure 2B,D). The FRET assay also showed that p-S129 promoted the aggregation rate of α-syn and enhanced the ability of PFF to induce monomer aggregation [53].

OW and OP were highly stable, strongly suggesting that most of the α-syn monomers were covalently cross-linked in the generated oligomeric structure. In contrast, large parts of PFF and pPFF dissociated into monomeric proteins after treatment with SDS (Figure 3A–D). To test the compactness of the four types of α-syn aggregates, the proteins were treated with PK, a broad-based protease that cleaves accessible residues with no amino acid side chain specificity. So, treatment with PK provided information about the structural features of the OW, OP, PFF, and pPFF. The results showed that PFF and pPFF were much more sensitive to PK treatment than OW and OP. Furthermore, OP and pPFF had more protein bands than OW and PFF, implying that PFF had more dynamic regions and OW was more stable, possibly due to the cross-links formed by ONE (Figure 3E,F). These findings indicate that although the ONE-induced α-syn aggregative structure is more compact and less accessible to PK digestion, ONE-induced α-syn oligomers have a higher degree of intermolecular crosslinking between His and Lys and between Lys and Lys, forcing hydrophilic amino acids to form a hydrophobic core [15]. This therefore explains how OW and OP are amorphous and more stable than PFF and pPFF. Not surprising, malondialdehyde-Lys modifications of α-syn were observed in the substantia nigra of PD patients, indicating that Lys residues are accessible for lipid peroxidation modifications [48]. Epitope mapping revealed that the N-terminal of α-syn (containing both the His residue and 11 of the 15 Lys residues) was hidden in ONE-induced α-syn aggregates [15]. Differently, the central hydrophobic NAC domain of α-syn has been shown to be a crucial region for the aggregation of α-syn [54,55]. Researchers reported that a 12-amino acid stretch (VTGVTAVAQKTV) in the NAC of human α-syn is necessary and sufficient for its fibrillization [56]. The formation of PFF is thought to be achieved through NAC regions. Solid-state NMR structure of a pathogenic fibril of α-syn showed that PFF had amyloid features, including parallel in-register β-sheets and hydrophobic core residues. In addition, PFF had diverse structural features: an intermolecular salt bridge, a glutamine ladder, close backbone interactions involving small residues, and several steric zippers stabilizing a novel, orthogonal Greek-key topology. Previous studies showed that different strains of α-syn fibrils possess different secondary structures, levels of toxicity, and propagation properties, which may be due to different exposed regions in their fibril structures [57].

Among the other characteristics, the ability to induce toxicity in the cells is an important feature of four aggregates. To investigate the different toxic properties of ONE- or agitation-induced α-syn and p-α-syn aggregates, the effect on the viability of SH-SY5Y cells and the presence of p-α-syn inclusions were assessed. The results of the MTT assay demonstrated that PFF and pPFF decreased cell viability earlier than OW and OP. Notably, pPFF were more toxic than OW, OP, and PFF (Figure 4). In a previous study, ONE-induced α-syn oligomers were toxic [15]. Recently, much research has been directed at the extracellular effects and p-α-syn inclusions in cells, suggesting that the pathology of Lewy bodies may spread among neurons. The spread of pathologic α-syn in the brain was assessed by phosphorylation of Ser129 [40]. Our results showed that pPFF had more p-α-syn inclusions than PFF, OP, and OW (Figure 5). OW, OP, PFF, and pPFF as seeds could induce the aggregative p-α-syn. For OW, it could influence mitochondria’s structure, function, and defensive ability [58]. OW further causes cell damage and produces ROS, forming a vicious cycle and producing more lipid superoxidation and p-α-syn aggregates. This is the first report of differences in cell toxicity of ONE- and agitation-induced α-syn and p-α-syn aggregates. However, it remains unclear whether the phosphorylated form of the aggregates is the cause or the result of the disease. It is possible that the spread of aggregated α-syn among cells plays a role in toxicity. In this study, an additive aggregate model, rather than a propagation model, was used to compare aggregation toxicity.

α-Syn and p-α-syn not only cause disease as toxic proteins, but also are potential biomarkers to detect disease, particularly for aggregates of p-α-syn [59,60]. Many studies have examined p-α-syn in cerebrospinal fluid, plasma, and red blood cells. However, at present, there is no unified standard, thus p-α-syn is not appropriate as a diagnostic or prognostic biomarker. The results of the present study showed that OW and OP were comparatively more stable. So, the sensitivities of OW, OP, PFF, and PPFF for standard antigen detection were compared. The results showed that the OP- and OW-specific ELISA had higher sensitivity for protein detection (Figure 6). Therefore, the OP- and OW-specific ELISA could be used to detect aggregate proteins in the plasma of Thy1-α-syn Tg mice to reflect pathogenesis (Figure 7). The results showed that these content changes of α-syn and p-α-syn were consistent with the movement disorders of mice, so the ELISA detection method using OW and OP could reflect the disease process of mice. Our results proved that OW and OP are promising for ELISA detection. This method can then also be used to detect the levels of α-syn and p-α-syn in human plasma in the future. Therefore, the OP- and OW-specific ELISAs would have greater value for diagnosis of disease.

There were some limitations to this study that should be addressed. First, we did not explore why aggregates induced by physical agitation were more toxic than those induced by ONE. The morphological and conformational properties of aggregates maybe explain their differential toxicity. The agitation-induced aggregates mainly depended on the NAC domain. So, the C-terminal of α-syn was exposed, which can cause damage to the membrane structure and oxidative stress [58,61]. However, the aggregates caused by ONE have a denser structure, so less C-terminal is exposed and bonded to the membrane, resulting in less oxidation and relatively low toxicity. Second, the reasons why p-α-syn is more sensitive to aggregation and is more toxic than non-p-α-syn were not investigated. Ser129 phosphorylation was demonstrated to alter conformation of the α-syn [62]. The p-α-syn demonstrated rapid and efficient conversion with the formation of various beta-hairpins. The NAC domain was simultaneously bound by two residues at its ends, thereby inducing the formation of the loops [63]. Ser129 phosphorylation increased membrane binding and cellular uptake of pPFF in vitro [64]. Additionally, some studies have shown that p-α-syn, as an important post-translational modification of α-syn, plays an important role in the origin of diseases [65].

## 5. Conclusions

The current study is the first to report that OW, OP, PFF, and pPFF had distinct morphological, biochemical, and functional properties. A summary of the characters presented in this manuscript can be found in Table 1. With this information, PFF and pPFF would be a reliable model of pathological protein aggregation in PD research and OW, and OP could be used as antigens for disease detection and diagnosis. Importantly, our experiments highlight the importance of phosphorylation in the events of α-syn. Increasing the robustness of the α-syn model and stability of aggregates should hopefully provide a new avenue to improve understanding of pathological mechanisms, diagnostic biomarkers, new disease-modifying treatments for PD, and other synucleinopathies.

## Figures and Tables

**Table 1:** Properties of ONE- and agitation-induced α-syn and p-α-syn aggregates.

Species	OW	OP	PFF	pPFF
Incubation times	18 h	18 h	7 days	7 days
Fibrils width	5.828 ± 2.368 nm	7.597± 3.241 nm ^(^*^)^	4.017± 2.205 nm ^(#)^	5.851 ± 2.128 nm ^(^*^,$)^
ThT (ratio to control)	6.762 ± 0.9715	18.47 ± 0.4895 ^(^****^)^	35.78 ± 2.35 ^(####)^	46.76 ± 1.568 ^(^****^,$$$$)^
p-α-syn aggregate speed (ratio to α-syn)	1	1.409 ± 0.016 (18 h) ^(^***^)^	1	1.497 ±0.111 (7Days) ^(^***^)^
Density (ratio to OW)	1	1.119 ± 0.1126	0.733 ± 0.0469 ^(#)^	0.8981 ± 0.09846 ^($)^
SDS-Stability of aggregates (ratio to monomeric protein)	13.13 ± 2.239	7.143 ± 2.284	2.335 ± 0.3494 ^(###)^	3.201 ± 1.068 ^($)^
PK Resistance	++	+++	+	++
Cell toxic sensibility times	6 h	3 h	1 h	1 h
Prefer application	Antigens for ELISA	Antigens for ELISA	Seeds for model	Seeds for model

Abbreviations: OW, ONE-induced WT α-syn aggregates; OP, ONE-induced p-α-syn aggregates; PFF, agitation-induced α-syn preformed fibrils; pPFF, p-α-syn preformed fibrils. * *p* < 0.05, *** *p* < 0.001, **** *p* < 0.0001 vs. OW and PFF. ^#^ *p* < 0.05, ^###^ *p* < 0.001, ^####^ *p* < 0.0001 vs. OW. ^$^ *p* < 0.05 or ^$$$$^ *p* < 0.0001 vs. OP.

## Data Availability

The data presented in this study are available in this article.
